# The Moderating and Mediating Role of Psychological Resilience in the Relationship Between Borderline Personality Symptoms and Suicidal Ideation Among University Students

**DOI:** 10.3390/ejihpe16040053

**Published:** 2026-04-16

**Authors:** Emadeldin M. Elsokkary, Abd elmureed Abd elgaber Kaseem, Abdulrahman Suliman Alnamlah

**Affiliations:** Department of Psychology, College of Social Sciences, Imam Mohammad Ibn Saud Islamic University (IMSIU), Riyadh 13317, Saudi Arabia; aamkasm@imamu.edu.sa (A.e.A.e.K.); asnamlah@imamu.edu.sa (A.S.A.)

**Keywords:** borderline personality symptoms, psychological resilience, suicidal ideation, mediation, moderation, PROCESS

## Abstract

**Objective**: This study examined psychological resilience (PR) as a potential moderator and mediator of the association between borderline personality symptoms (BPS) and suicidal ideation (SI) among university students. **Method**: A cross-sectional design was used with (*N* = 257) university students. Moderation and mediation were tested in separate, theory-guided models using the PROCESS macro for SPSS, version 28. The moderation model (Model 1) and the mediation model (Model 4) were estimated with heteroskedasticity-consistent standard errors (HC3). In the adjusted analyses, sex, age, previous psychological consultation, previous psychotropic medication use, and family history of mental illness were entered as covariates. The indirect effect was evaluated using percentile bootstrap confidence intervals based on (5000) resamples. **Results**: BPS was positively correlated with SI, whereas PR was negatively correlated with both BPS and SI. In the adjusted moderation model, BPS was positively associated with SI (b = 0.118, *p* < 0.001) and PR was negatively associated with SI (b = −0.204, *p* = 0.048), but the interaction term was not significant (b = −0.001, *p* = 0.820) with negligible explained variance (ΔR^2^ = 0.0003). In the adjusted mediation model, BPS was significantly associated with lower PR (a: b = −0.135, *p* < 0.001), and PR was associated with lower SI while controlling for BPS and the covariates (b: b = −0.216, *p* = 0.028). The total effect of BPS on SI was significant (c: b = 0.146, *p* < 0.001), and the direct effect remained significant after including PR (c′: b = 0.117, *p* < 0.001). The indirect effect was significant (ab = 0.029; 95% bootstrap CI [0.005, 0.061]). **Conclusions**: Psychological resilience did not moderate the association between BPS and suicidal ideation, but it showed a statistically significant indirect association consistent with the proposed mediation model. Higher BPS were associated with lower resilience, which in turn was associated with higher suicidal ideation. These findings suggest that resilience-related targets may complement interventions addressing core BPS-related risk processes, while the cross-sectional design precludes causal conclusions.

## 1. Introduction

Suicide is a universal human phenomenon that has accompanied human existence since its earliest beginnings, manifesting in varying degrees across different societies, cultural contexts, and temporal and geographic settings. In contemporary contexts, suicide is no longer regarded merely as an isolated individual behavior, but rather as a pressing global health and social issue, representing one of the leading causes of premature death across diverse populations, in addition to its profound psychological, social, and economic impacts on individuals, families, and societies at large (Altwaijri et al., 2024). Reports from the World Health Organization indicate that more than 720,000 people die by suicide every year; specifically, an estimated 727,000 people died by suicide in 2021 (World Health Organization, 2025). Recent WHO statistics highlight the heightened risk of suicide among youth, identifying it as the (third) leading cause of death among individuals aged (15–29) globally in (2021) (World Health Organization, 2025).

Within this context, suicidal ideation emerges as a critical early indicator for understanding suicidal behavior. It is defined as a cognitive process in which an individual contemplates ending their life without taking action, marking a pivotal preparatory stage in the developmental trajectory of suicidal behavior and guiding early, effective preventive interventions (Timpka et al., 2021; Arafat et al., 2025).

The World Health Organization (WHO) emphasizes the importance of early identification and timely support for people at risk of suicide and self-harm as a core component of suicide prevention (World Health Organization, 2019). The American Association of Suicidology describes suicidal ideation as thoughts, fantasies, or ideas related to ending one’s life, ranging from fleeting thoughts to detailed plans, and distinguishes between passive ideation (thoughts about death or a desire to die without a specific plan) and active ideation (suicidal thoughts accompanied by planning or intent) (American Association of Suicidology, 2023). Because suicidal ideation is a significant risk factor for subsequent suicide attempts and suicide deaths, it represents a key target for risk detection and preventive intervention, particularly in contexts where rapid access to specialized mental health care may be limited.

Suicidal ideation is particularly salient among university students, given the transitional nature of this developmental stage from adolescence to early adulthood, coupled with academic, psychological, and social stressors that may amplify suicidal thinking (Santos et al., 2017). This period is characterized by increasing autonomy, identity consolidation, academic performance pressure, and changes in social and family roles, which may interact with psychological vulnerabilities. Suicidal ideation can emerge across ages and backgrounds, often in conjunction with psychiatric conditions, but it is also observed in community samples under cumulative stress exposure (Rizvi et al., 2024). Risk factors contributing to suicidal ideation include previous suicide attempts, chronic illnesses, psychosocial stressors, trauma exposure, and weak social support. The underlying mechanisms involve complex interactions among neurobiological, genetic, and environmental factors that influence mood regulation and impulse control, thereby increasing the likelihood that suicidal thinking progresses to behavior (Nikmehr et al., 2025).

Among psychological factors, personality pathology—particularly borderline personality symptoms (BPS)—is among the most significant correlations of elevated suicide risk. In the present study, borderline personality symptoms refer to dimensional borderline features assessed with the BSL-23, including affective instability, impulsivity, and interpersonal dysregulation, rather than to a categorical diagnosis alone (American Psychiatric Association, 2013; Bohus et al., 2009). Longitudinal evidence indicates that affective instability associated with BPS is a strong predictor of suicidal ideation and behavior, whereas the contribution of major depressive disorder appears less consistent in this specific context (Links et al., 2007). Contemporary evidence further confirms the robust association between BPS and heightened suicidal thoughts and attempts across age groups and clinical statuses (Lak et al., 2025). In addition, suicidal risk in individuals with BPS is closely linked to emotion dysregulation, impulsivity, maladaptive coping, and recurrent interpersonal crises, while comorbid conditions may further intensify severity and recurrence of suicidal behaviors (Bitran et al., 2025; Tennenhouse et al., 2025). Recent work also suggests that subthreshold borderline traits may amplify suicidal thoughts over time, and that both borderline symptomatology and prior suicidal behavior may independently predict later suicidal outcomes (Carpita et al., 2025; Aouidad et al., 2020). From a classificatory perspective, BPS may confer additional risk for suicidal ideation beyond major depressive disorder, although the psychological resources that may attenuate this risk remain insufficiently understood (Moran et al., 2012; Brent et al., 1993).

In parallel with this risk-focused literature, recent scholarship has increasingly emphasized protective factors that may reduce vulnerability to suicidal ideation. Among these, psychological resilience has emerged as a central construct in positive psychology, broadly reflecting the capacity to cope effectively with adversity and adapt to difficult life experiences (Gupta et al., 2015; American Psychological Association, 2018). Prior research suggests that resilience is generally associated with lower suicidal ideation and may operate as a protective process in suicide prevention (Sher, 2019; Siegmann et al., 2018). It has also been conceptualized as a potential buffer against the effects of psychological stressors and vulnerability factors relevant to suicidality, including emotional instability and impulsivity (Massaro, 2015). At the same time, resilience may extend beyond a direct protective association by functioning through mediating and moderating processes, potentially involving coping resources, self-efficacy, and adaptive adjustment mechanisms (Liu et al., 2017; Caredda et al., 2024).

This issue appears especially relevant in the context of borderline traits among youth and university student populations. Existing evidence suggests that resilience may partly account for the link between personality-related vulnerabilities and suicidal ideation, sometimes alongside other cognitive-emotional processes such as symptom-focused rumination. For example, students with borderline traits have been shown to report higher levels of suicidal ideation and suicide attempts, with resilience emerging as a protective factor and an indirect pathway in the association between borderline traits and suicidality (He et al., 2025). Similarly, resilience has been reported as a mediator between personality dimensions and suicidal ideation in younger populations, supporting its potential explanatory role within personality–suicidality pathways (Che et al., 2022). At the same time, empirical work examining resilience as both a mediator and a moderator in the association between BPS and suicidal ideation remains limited, particularly in university populations.

This gap is especially important in the Arab context, including Saudi Arabia, where research and surveillance on suicidal behavior remain relatively limited. Stigma and religious, social, and legal sensitivities may discourage disclosure and help-seeking, while variation in surveillance quality may contribute to under-recording and uncertainty in official estimates (Erlangsen et al., 2024; Arafat et al., 2021). Available indications suggest elevated suicide-related risk among youth and adolescents aged 15–24, with notable gender differences in attempts and completions (Altwaijri et al., 2024). Thus, there is a clear need for integrated models that examine both vulnerability processes and potentially modifiable protective factors in university students within this sociocultural context.

Accordingly, the present study examined psychological resilience as both a potential mediator and a potential moderator of the association between BPS and suicidal ideation among university students. These two roles address different but complementary questions. As a mediator, resilience may help explain whether part of the observed association between borderline symptoms and suicidal ideation is statistically consistent with an indirect pathway involving coping resources and self-regulation. As a moderator, resilience may clarify whether the strength of the BPS–SI association varies across levels of resilience. Examining these roles may help refine theory, identify clinically relevant and mechanism-informed targets, and strengthen prevention efforts for youth at elevated suicide risk.

## 2. Materials and Methods

### 2.1. Study Design

This study used a quantitative, cross-sectional, non-experimental design to examine the relationships among Borderline Personality Symptoms (BPS), Suicidal Ideation (SI), and Psychological Resilience (PR) among university students, with a particular focus on the potential mediating and moderating roles of psychological resilience in the association between Borderline Personality Symptoms and Suicidal Ideation. Data were collected at a single point in time. Although causal conclusions cannot be drawn, this design is suitable for testing theory-guided models of mediation and moderation in terms of statistical associations and interaction effects. Accordingly, any mediation findings in the present study should be interpreted as consistent with the proposed theoretical model rather than as evidence of temporal or causal ordering.

The moderation and mediation analyses were conducted as separate, theory-guided models addressing distinct questions about resilience, rather than as simultaneous claims of temporal or causal ordering.

### 2.2. Ethical Approval and Consent

Ethical approval was obtained from the Research Ethics Committee at Imam Mohammad Ibn Saud Islamic University (IMSIU). Participation was voluntary, and electronic informed consent was obtained. Responses were anonymous, and participants could withdraw at any time. Procedures complied with the Declaration of Helsinki. Because the survey was anonymous, individual emergency follow-up could not be initiated for participants reporting elevated suicidal ideation. To address this ethically, all participants were provided at the end of the survey with mental health support information and guidance on how to seek professional help if needed.

### 2.3. General Characteristics of Study Participants

A total of (257) undergraduate students participated in this study. Participants were recruited from multiple colleges within a public university via an online survey disseminated through official university channels and classroom announcements. Participants were recruited using convenience sampling from the general university student population. Participation was voluntary. Eligible participants were undergraduate students enrolled at the study university, aged 18 years or older, able to complete the Arabic online survey, and willing to provide electronic informed consent. The sample was drawn from the general university student population rather than a clinical population. The electronic survey required responses to all items before submission; therefore, all analyzed questionnaires were complete. The mean age of participants was (19.8) years (SD = (1.11)). Table 1 presents the demographic characteristics of the sample.

### 2.4. Materials

**Borderline Symptom List-23** (BSL-23; Bohus et al., 2009). The BSL-23 is a widely used self-report instrument designed to assess the severity of borderline personality symptoms (BPS). In the present study, the brief Arabic version of the scale was employed. The measure consists of 23 items rated on a 5-point Likert scale ranging from 0 (not at all) to 4 (very strong). The scale is typically treated as unidimensional, reflecting a single underlying factor. The BSL-23 was selected because of its well-established psychometric utility and its coverage of core borderline features, including emotional dysregulation and impulsivity. Its brevity enhances feasibility for both research and clinical use, and its items provide a concise assessment of traits commonly associated with borderline pathology. The Arabic version used in the present study was adopted from the Arabic translation and adaptation reported by Abu Zeid (2017). Abu Zeid (2017) described translation of the brief borderline symptom measure into Arabic, expert review, back-translation into English, and verification of translation adequacy. In that study, item-total correlations ranged from 0.79 to 0.90, criterion-related validity reached 0.671, and reliability coefficients were high across methods, including 0.90 for test–retest reliability, 0.89 for split-half reliability, 0.89 using the Guttman formula, and 0.87 for Cronbach’s alpha. In addition, more recent Saudi research by Al-Zunaidi and Al-Halabi (2024) used the Arabic BSL-23 and provided additional psychometric support for its use in the Saudi context. In the current sample, the Arabic BSL-23 demonstrated excellent internal consistency (Cronbach’s α = 0.947; McDonald’s ω = 0.946), supporting its reliability for use in the present study.

**The 10-item Connor–Davidson Resilience Scale** (CD-RISC-10; Campbell-Sills & Stein, 2007) is a self-report instrument developed to assess individuals’ capacity to cope with adversity. The CD-RISC-10 is a short form derived from the original 25-item CD-RISC (Connor & Davidson, 2003) and has been widely used to evaluate psychological resilience across diverse populations. Each item is rated on a 5-point Likert scale ranging from 0 (not true at all) to 4 (true nearly all of the time), yielding total scores from 0 to 40, with higher scores indicating greater resilience. In the present study, the scale was administered in Arabic. The Arabic version used in the present study was adopted from the Arabic translation reported by Toma et al. (2017), who described a systematic translation process involving double forward translation, reconciliation, independent back-translation, consultation with the instrument developer, and cognitive testing with six Arabic-speaking individuals. This Arabic version was also used in the Saudi context by Rayani et al. (2024) among university nursing students in Saudi Arabia, with satisfactory psychometric support, including internal consistency (Cronbach’s α = 0.84), reported in that sample. In the current sample, the CD-RISC-10 demonstrated excellent internal consistency (Cronbach’s α = 0.877; McDonald’s ω = 0.878), supporting its reliability for use in the present study.

**The Suicidal Ideation Scale** (SIS; Al-Didane, 2015) is a (14)-item measure designed to assess suicidal thinking. The scale comprises two subscales: a family dimension (7 items: 1, 3, 5, 7, 9, 11, 13) and a psychological dimension (7 items: 2, 4, 6, 8, 10, 12, 14). Items are rated on a three-point Likert scale (Yes = 3, Somewhat = 2, No = 1), with higher scores indicating greater suicidal ideation (total score range: 14–42). In the current sample (*N* = 257), internal consistency was excellent for the total scale (Cronbach’s α = 0.893; McDonald’s ω = 0.891). Reliability for the family subscale was high (α = 0.868; ω = 0.874), and reliability for the psychological subscale was acceptable to good (α = 0.803; ω = 0.802), supporting the scale’s use for research purposes in the present study.

## 3. Results

### 3.1. Preliminary Analyses

Table 2 presents descriptive statistics and Pearson correlations among borderline personality symptoms (BPS), psychological resilience (PR), and suicidal ideation (SI) in the study sample (*N* = 257). BPS was positively correlated with SI, whereas PR was negatively correlated with both BPS and SI.

### 3.2. Moderation Analysis (PROCESS Model 1, HC3)

To test whether psychological resilience (PR) moderates the association between BPS and SI, a moderation model was estimated using the PROCESS macro for SPSS (Model 1; Hayes, 2018) with heteroskedasticity-consistent standard errors (HC3). BPS and PR were mean-centered prior to forming the interaction term (BPS × PR). Sex, age, previous psychological consultation, previous psychotropic medication use, and family history of mental illness were entered as covariates. Given evidence of heteroskedasticity (Breusch–Pagan test *p* < 0.001), HC3 robust standard errors were used for inference. HC3 was selected because it provides heteroskedasticity-robust inference and is commonly recommended for OLS-based models when heteroskedasticity is detected, helping to yield more reliable standard errors, confidence intervals, and significance tests (Hayes & Cai, 2007; Long & Ervin, 2000). Multicollinearity was not a concern (all VIFs < 1.70). Although BPS and PR were moderately correlated (r = −0.49), the low VIF values indicate that the two constructs were related but not collinear to an extent that would threaten model estimation.

A post hoc sensitivity analysis was conducted for the interaction term using a fixed-model multiple regression R^2^-increase framework. With *N* = 257, α = 0.05, power = 0.80, one tested predictor, and eight total predictors, the present moderation model was powered to detect an interaction effect of at least f^2^ = 0.032. The observed interaction effect in the current study (ΔR^2^ = 0.0003) was substantially smaller than this threshold.

As shown in Table 3, BPS was positively associated with SI (b = 0.118, *p* < 0.001), and PR was negatively associated with SI (b = −0.204, *p* = 0.048), even after controlling for the covariates. However, the BPS × PR interaction was not statistically significant (b = −0.001, *p* = 0.820), and the increment in explained variance due to the interaction remained negligible (ΔR^2^ = 0.0003).

### 3.3. Mediation Analysis (PROCESS Model 4, HC3; 5000 Bootstrap Samples)

To test whether psychological resilience (PR) mediates the relationship between BPS and SI, a simple mediation model was estimated using the PROCESS macro for SPSS (Model 4; Hayes, 2018). Sex, age, previous psychological consultation, previous psychotropic medication use, and family history of mental illness were entered as covariates in the mediator and outcome models. Because heteroskedasticity was present (Breusch–Pagan test *p* < 0.001), HC3 robust standard errors were used. This approach was used to improve the robustness of statistical inference under heteroskedasticity. The indirect effect was evaluated using percentile bootstrap confidence intervals based on 5000 resamples.

Results in Table 4 show that BPS was significantly associated with lower PR (path a: b = −0.135, *p* < 0.001). In turn, PR was significantly associated with lower SI while controlling for BPS and the covariates (path b: b = −0.216, *p* = 0.028). The total effect of BPS on SI remained significant (path c: b = 0.146, *p* < 0.001), and the direct effect also remained significant after including PR (path c′: b = 0.117, *p* < 0.001). The indirect effect through PR was statistically significant (ab = 0.029; 95% bootstrap CI [0.005, 0.061]), indicating that PR carried a small but reliable portion of the association between BPS and SI even after adjustment for the covariates. The proportion mediated was 0.200 (19.97%), with a 95% bootstrap CI [0.038, 0.458], suggesting that approximately one-fifth of the total association operated indirectly through PR. Taken together, this pattern is consistent with partial mediation, because the indirect effect through PR was statistically significant while the direct effect of BPS on SI remained significant after including the mediator.

**Figure 1:**
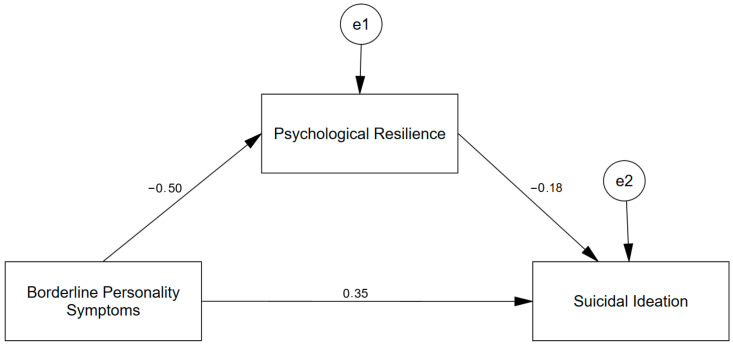
Adjusted indirect association model illustrating psychological resilience (PR) in the association between borderline personality symptoms (BPS) and suicidal ideation (SI). *Note*. Standardized path coefficients from the adjusted mediation model are displayed on the arrows. Covariates included in the statistical model are omitted from the figure for visual clarity. Unstandardized estimates from PROCESS (HC3) are reported in Table 4.

A path diagram illustrating the adjusted indirect association model is provided in Figure 1 (standardized estimates displayed for clarity).

## 4. Discussion

This study examined psychological resilience (PR) as both a moderator and a mediator of the association between borderline personality symptoms (BPS) and suicidal ideation (SI) among university students. Using robust (HC3) inference to address heteroskedasticity, the findings yielded a clear pattern: BPS was positively associated with SI, PR was negatively associated with SI, and the BPS × PR interaction was not significant. In contrast, the mediation analysis revealed a statistically significant indirect association consistent with the proposed theoretical model, such that higher BPS were associated with lower resilience, which in turn was associated with higher suicidal ideation, while the direct association between BPS and SI remained significant. This combination—a non-significant moderation effect alongside a significant indirect association—offers a theoretically meaningful account of how resilience may relate to suicidal thinking in the context of borderline symptoms in a university population, while remaining subject to the limitations of cross-sectional data.

### 4.1. Why Did Resilience Emerge as a Mediator but Not a Moderator?

At face value, it may appear surprising that resilience was involved in a significant indirect association within the BPS–SI relationship (mediation) but did not buffer it (moderation). However, these results are conceptually compatible because mediation and moderation address different scientific questions. In the present cross-sectional study, the mediation analysis should be understood as identifying a statistically significant indirect association consistent with the proposed theoretical model (BPS → PR → SI), whereas moderation concerns whether the magnitude of the BPS → SI association differs across levels of resilience. Thus, resilience may plausibly function as a psychological resource that is lower among individuals with elevated borderline symptoms and is also associated with suicidal ideation, without necessarily operating as a protective “shield” that weakens the BPS–SI slope.

A second interpretation is that resilience in this context may be more closely linked to the pattern of associations surrounding borderline symptom severity than to a buffering role against its effects. BPS symptomatology is characterized by affective instability, emotion dysregulation, impulsivity, and recurrent interpersonal crises—processes that can consume and undermine coping resources over time (Links et al., 2007; Sher, 2019). From a resource-based account (e.g., Conservation of Resources theory), chronic dysregulation and recurrent stressors may be associated with resource depletion, making resilience less an external moderator and more a psychological asset that becomes compromised as symptom severity increases (Hobfoll, 1989). In that scenario, lower resilience may represent one statistically supported indirect link in the observed association between borderline symptoms and suicidal thinking, rather than a confirmed temporal mechanism.

Third, the absence of moderation may reflect both substantive and statistical considerations. Substantively, the BPS–SI association may be relatively strong and uniform in a student sample, such that resilience reduces overall SI (main effect) but does not materially alter the gradient linking borderline symptoms to suicidal thinking. Statistically, interaction effects in observational data are often small and require substantial variability and power to detect reliably. Restricted range in resilience and related protective resources is plausible in university populations relative to clinical samples, potentially limiting the likelihood of detecting BPS×PR interaction effects even when main effects are robust (McGee et al., 2018).

Finally, the coexistence of a significant direct effect and a significant indirect effect supports a nuanced interpretation: resilience explains a reliable but modest portion of the BPS–SI association, while a substantial direct pathway remains, suggesting additional mechanisms such as rumination, interpersonal stress sensitivity, comorbid depressive symptoms, hopelessness, and impulsive responding may also connect borderline symptom severity to suicidal ideation (Carpita et al., 2025; Moran et al., 2012; Brent et al., 1993). The size of the indirect effect also supports a balanced interpretation. In the adjusted mediation model, approximately one-fifth of the total association between BPS and suicidal ideation was statistically consistent with the indirect pathway through resilience. This suggests that resilience is neither a trivial correlate nor a complete explanation of the BPS–SI association. Rather, it appears to represent one meaningful but partial pathway within a broader risk structure in which additional cognitive-affective and contextual mechanisms are also likely to contribute.

### 4.2. Integration with Prior Literature

The present mediation finding is consistent with a growing body of work showing that resilience is inversely associated with suicidal ideation and may operate as a protective process within broader risk pathways (Sher, 2019; Siegmann et al., 2018).

Importantly, prior evidence in youth and student samples indicates that resilience can partially account for how borderline traits relate to suicidality, often in conjunction with other cognitive-emotional processes such as symptom-focused rumination (He et al., 2025). Similarly, resilience has been identified as a mediator between personality dimensions and suicidal ideation in younger populations (Che et al., 2022), supporting the plausibility of resilience as a mechanism within personality–suicidality pathways.

At the same time, the null moderation result is also defensible in light of the broader literature. Although resilience has been conceptualized as a buffer under stress and adversity, moderation effects are frequently context-dependent and may be more likely to emerge when resilience interacts with proximal stressors (e.g., acute stress exposure, low social support, coherence/meaning systems) rather than with pervasive, trait-like vulnerability patterns such as borderline symptomatology (Caredda et al., 2024; McGee et al., 2018). In practical terms, resilience may reduce overall suicidal ideation and carry part of the pathway from BPS to SI (mediation), yet still not be sufficient—within the observed range—to modify the strength of the borderline symptom–suicidal thinking link in a statistically detectable manner. This pattern is consistent with prior work suggesting that resilience may be associated with lower overall suicidal ideation without necessarily moderating the effect of broader vulnerability patterns such as BPS, particularly when moderation effects depend on contextual or proximal stress-related conditions (Caredda et al., 2024; McGee et al., 2018).

These findings also converge with evidence that suicidal risk in BPS reflects multidimensional pathways, including emotion regulation deficits and comorbid conditions that can amplify risk (Bitran et al., 2025; Tennenhouse et al., 2025). The present pattern suggests that resilience is one meaningful piece of a broader etiological mosaic rather than a comprehensive protective moderator that offsets borderline-related risk.

These findings should also be interpreted in light of the Arab and Saudi context highlighted in the introduction. In settings where stigma, religious sensitivities, and social concerns may discourage disclosure of suicidal thoughts and delay help-seeking, the observed associations between BPS, resilience, and suicidal ideation may have particular practical relevance. The relative scarcity of systematic research and surveillance on suicidal behavior in Arab populations further increases the importance of identifying both vulnerability factors and potentially modifiable protective processes in university samples. In this context, the present findings contribute not only to the broader literature on borderline symptoms and suicidal ideation, but also to a still-limited regional evidence base that may inform culturally sensitive prevention, screening, and early support efforts.

### 4.3. Clinical and Practical Implications

The results have implications for clinical practice and campus mental health services. First, the robust direct association between BPS and suicidal ideation underscores that suicide risk assessment should remain proactive when borderline symptoms are elevated, even when resilience is considered. Second, the mediation pathway supports the clinical value of resilience-related targets (e.g., adaptive coping, problem solving, regulation skills), because resilience-oriented interventions may help address one indirect pathway consistent with the proposed model linking BPS symptoms to suicidal thinking. However, the lack of moderation suggests that resilience-building alone may be insufficient as a stand-alone buffer, and interventions should also address core BPS-related processes directly (e.g., emotion dysregulation, distress intolerance, interpersonal instability, and crisis planning). At the same time, because the BPS × PR interaction was not statistically significant, the present findings do not support the conclusion that resilience buffers the BPS–SI association differently across levels of symptom severity. This aligns with evidence-based approaches such as Dialectical Behavior Therapy, which targets suicidal risk while building distress tolerance and emotion regulation skills.

More specifically, the present findings suggest that university-based mental health responses should not rely solely on generalized resilience promotion. Instead, students with elevated BPS may benefit from layered intervention strategies that combine proactive suicide risk screening with targeted support for emotion regulation, distress tolerance, adaptive problem solving, interpersonal functioning, and safety planning. In practical terms, resilience-informed interventions may be most useful when embedded within broader support pathways that include confidential screening procedures, timely referral to counseling or psychiatric services, and brief skills-based programs adapted for students at elevated risk. Because the direct association between BPS and suicidal ideation remained significant even after accounting for resilience, interventions should address both resilience-related resources and core borderline-related processes rather than assuming that resilience enhancement alone will be sufficient. The resilience-related targets suggested by the present findings include adaptive coping, emotion regulation and tolerance of negative affect, flexibility in responding to stress, and personal competence or self-efficacy when facing adversity.

### 4.4. Limitations and Directions for Future Research

Several limitations warrant caution, particularly regarding causal interpretation. First, the study was cross-sectional; therefore, the mediation findings should be interpreted as consistent with an indirect association under the proposed theoretical model rather than as evidence of temporal or causal effects. Longitudinal designs (e.g., prospective mediation, cross-lagged models) are needed to examine whether borderline symptoms are linked to subsequent reductions in resilience and whether such reductions precede increases in suicidal ideation over time. Although alternative directional models are conceivable, such models cannot resolve the issue of temporal precedence in the absence of longitudinal data. Second, because the study relied on self-report measures collected at a single time point, the observed associations may have been influenced to some extent by shared method variance. Future studies would benefit from incorporating multi-method assessment strategies, such as clinical interviews, informant reports, or behavioral indicators. Third, the relatively modest sample size and the use of a convenience sample drawn from a general university student population may limit the representativeness and generalizability of the findings. Replication in broader community and clinical samples is needed, particularly because interaction effects may be more detectable when resilience variability is wider. Fourth, although prior Arabic adaptation work and supporting psychometric evidence were available for the instruments used in this study, further cross-cultural validation in diverse Arabic-speaking samples remains desirable. Finally, given the remaining direct effect, future work should examine richer explanatory models that integrate proximal cognitive-affective variables (e.g., rumination, hopelessness), psychiatric comorbidity, and contextual moderators (e.g., trauma exposure, social support), and test whether resilience moderates effects of acute stressors more readily than the trait-like influence of borderline symptoms.

The anonymous design enhanced privacy and may have facilitated disclosure, but it also limited the possibility of individualized follow-up for participants reporting elevated suicidal ideation.

## 5. Conclusions

Overall, psychological resilience was involved in a statistically significant indirect association between borderline personality symptoms and suicidal ideation, while not operating as a moderator that changed the strength of the BPS–SI association. This pattern is conceptually coherent: resilience may represent a psychological resource that is lower in the context of elevated BPS and is also associated with suicidal ideation, yet may not be strong enough—or sufficiently variable in this student sample—to buffer the overall BPS-related risk gradient. In practical terms, resilience-oriented efforts in university settings may be most useful when they target adaptive coping, emotion regulation, tolerance of negative affect, flexibility under stress, and personal competence, rather than relying on generalized resilience promotion alone.

## Figures and Tables

**Table 1:** General characteristics of 257 study participants.

Characteristics	*N* (%)
**Sex**	
Male	147 (57.1%)
Female	110 (42.8%)
**Academic Level**	
Levels 1–3	85 (33.1%)
Levels 4–5	162 (63.0%)
Levels 6–7	6 (2.3%)
Level 8	4 (1.6%)
**Academic Specialization**	
Scientific Colleges	115 (44.7%)
Humanities Colleges	142 (55.2%)
**Previous Psychological Consultation**	
Yes	23 (8.9%)
No	234 (91.1%)
**Previous Psychotropic Medication Use**	
Yes	20 (7.8%)
No	237 (92.2%)
**Family History of Mental Illness**	
Yes	77 (30.0%)
No	180 (70.0%)

**Table 2:** Descriptive statistics and correlations among study variables (*N* = 257).

Variable	M	SD	1	2	3
1. Suicidal Ideation (SI)	20.21	5.81	—		
2. Borderline Personality Symptoms (BPS)	43.86	17.49	0.53 **	—	
3. Psychological Resilience (PR)	36.04	4.72	−0.40 **	−0.49 **	—

*Note.* ** *p* < 0.01 (two-tailed).

**Table 3:** Moderation model examining associations of BPS, PR, their interaction, and covariates with suicidal ideation (PROCESS Model 1, HC3).

Variable	b	SE (HC3)	t	*p*	95% CI LL	95% CI UL
Constant	19.832	1.278	15.523	<0.001	17.328	22.336
BPS (X)	0.118	0.029	4.050	<0.001	0.061	0.175
PR (W)	−0.204	0.103	−1.981	0.048	−0.405	−0.002
BPS × PR	−0.001	0.006	−0.227	0.820	−0.012	0.010
Sex	0.100	0.567	0.176	0.860	−1.012	1.212
Age	−0.438	0.395	−1.110	0.267	−1.211	0.336
Previous psychological consultation	−1.313	1.660	−0.791	0.429	−4.566	1.941
Previous psychotropic medication use	4.077	1.988	2.051	0.040	0.180	7.974
Family history of mental illness	2.543	0.759	3.350	0.001	1.055	4.032

*Model summary.* R^2^ = 0.366, F(HC3)(8, 248) = 12.453, *p* < 0.001. Interaction test. ΔR^2^ = 0.0003, F(HC3)(1, 248) = 0.051, *p* = 0.821. Note. BPS and PR were mean-centered prior to forming the interaction term. Sex, age, previous psychological consultation, previous psychotropic medication use, and family history of mental illness were entered as covariates.

**Table 4:** Mediation of the association between BPS and suicidal ideation through psychological resilience, controlling for covariates (PROCESS Model 4, HC3; 5000 bootstrap samples).

**Model A (Outcome: PR; Path a)**						
**Variable**	**b**	**SE (HC3)**	**t**	***p***	**95% CI LL**	**95% CI UL**
Constant	41.059	1.482	27.713	<0.001	38.156	43.963
BPS (X)	−0.135	0.021	−6.309	<0.001	−0.177	−0.093
Sex	0.438	0.541	0.810	0.418	−0.622	1.498
Age	0.243	0.355	0.686	0.493	−0.452	0.939
Previous psychological consultation	1.718	1.463	1.174	0.240	−1.150	4.586
Previous psychotropic medication use	0.547	1.560	0.350	0.726	−2.511	3.604
Family history of mental illness	−0.995	0.714	−1.393	0.164	−2.394	0.405
*Model summary.* R^2^ = 0.263, F(HC3)(6, 250) = 11.424, *p* < 0.001.						
**Model B (Outcome: SI; Paths b and c′)**						
**Variable**	**b**	**SE (HC3)**	**t**	***p***	**95% CI LL**	**95% CI UL**
Constant	22.557	4.580	4.925	<0.001	13.580	31.534
BPS (X)	0.117	0.028	4.139	<0.001	0.062	0.172
PR (M)	−0.216	0.098	−2.200	0.028	−0.407	−0.024
Sex	0.093	0.562	0.166	0.868	−1.008	1.195
Age	−0.452	0.390	−1.159	0.246	−1.215	0.312
Previous psychological consultation	−1.307	1.644	−0.795	0.427	−4.530	1.915
Previous psychotropic medication use	4.084	1.964	2.079	0.038	0.234	7.934
Family history of mental illness	2.541	0.754	3.368	0.001	1.062	4.019
*Model summary.* R^2^ = 0.366, F(HC3)(7, 249) = 14.834, *p* < 0.001.						
**Total Effect Model (Outcome: SI; Path c)**						
**Variable**	**b**	**SE (HC3)**	**t**	***p***	**95% CI LL**	**95% CI UL**
Constant	13.708	1.595	8.595	<0.001	10.582	16.835
BPS (X)	0.146	0.023	6.407	<0.001	0.101	0.191
Sex	−0.001	0.576	−0.002	0.999	−1.130	1.128
Age	−0.504	0.413	−1.221	0.222	−1.313	0.305
Previous psychological consultation	−1.677	1.593	−1.053	0.292	−4.799	1.444
Previous psychotropic medication use	3.966	1.904	2.083	0.037	0.235	7.697
Family history of mental illness	2.755	0.759	3.631	<0.001	1.268	4.242
*Model summary.* R^2^ = 0.343, F(HC3)(6, 250) = 15.338, *p* < 0.001.						
**Indirect Effect (ab; Bootstrap)**						
**Indirect path**		**Effect**	**BootSE**		**BootLLCI**	**BootULCI**
BPS → PR → SI		0.029	0.014		0.005	0.061
Proportion mediated (ab/c)		0.200	0.109		0.038	0.458

*Note.* Indirect effects are evaluated using percentile bootstrap confidence intervals based on 5000 resamples. An indirect effect is considered statistically significant when the CI does not include zero. Sex, age, previous psychological consultation, previous psychotropic medication use, and family history of mental illness were included as covariates in the mediation models.

## Data Availability

The datasets generated and/or analyzed during the current study are available from the corresponding author on reasonable request.
